# Identification, 3D-Reconstruction, and Classification of Dangerous Road Cracks

**DOI:** 10.3390/s23073578

**Published:** 2023-03-29

**Authors:** Souhir Sghaier, Moez Krichen, Imed Ben Dhaou, Hela Elmannai, Reem Alkanhel

**Affiliations:** 1 Department of Science and Technology, College of Ranyah, Taif University, P.O. Box 11099, Taif 21944, Saudi Arabia; 2 Faculty of Computer Science and Information Technology, Al-Baha University, Al-Baha 65528, Saudi Arabia; 3 ReDCAD Laboratory, National School of Engineers of Sfax, University of Sfax, Sfax 3029, Tunisia; 4 Department of Computer Science, Hekma School of Engineering, Computing and Informatics, Dar Al-Hekma University, Jeddah P.O. Box 34801, Saudi Arabia; 5 Department of Computing, University of Turku, 20500 Turku, Finland; 6 Higher Institute of Computer Sciences and Mathematics, Department of Technology, University of Monastir, Monastir 5000, Tunisia; 7 Department of Information Technology, College of Computer and Information Sciences, Princess Nourah Bint Abdulrahman University, P.O. Box 84428, Riyadh 11671, Saudi Arabia

**Keywords:** image processing, crack detection, 3D reconstruction, machine learning, crack characterization, crack classification

## Abstract

Advances in semiconductor technology and wireless sensor networks have permitted the development of automated inspection at diverse scales (machine, human, infrastructure, environment, etc.). However, automated identification of road cracks is still in its early stages. This is largely owing to the difficulty obtaining pavement photographs and the tiny size of flaws (cracks). The existence of pavement cracks and potholes reduces the value of the infrastructure, thus the severity of the fracture must be estimated. Annually, operators in many nations must audit thousands of kilometers of road to locate this degradation. This procedure is costly, sluggish, and produces fairly subjective results. The goal of this work is to create an efficient automated system for crack identification, extraction, and 3D reconstruction. The creation of crack-free roads is critical to preventing traffic deaths and saving lives. The proposed method consists of five major stages: detection of flaws after processing the input picture with the Gaussian filter, contrast adjustment, and ultimately, threshold-based segmentation. We created a database of road cracks to assess the efficacy of our proposed method. The result obtained are commendable and outperform previous state-of-the-art studies.

## 1. Introduction

Governments throughout the world are concerned about road safety. According to the United Nations, around 1.35 million people are killed in road accidents each year [1]. Traffic safety is a multifaceted issue that is influenced by several factors. Road safety is influenced by infrastructure, cars, and road users, as detailed in [2]. Road condition is one of the top 10 causes of accidents in Saudi Arabia [3]. Road cracks, ruts, faulting, and punchout deteriorate road safety and driving comfort [4]. Road maintenance should be carried out at scheduled intervals. Road conditions are determined using laser technology.

The automatic processing of road pavements [5] is one of the areas that has benefited from the technological contribution in terms of image processing [6,7]. However, the specificity of the application complicates the task of extraction to arrive at very precise solutions adapted to the nature of the road surfaces examined. These surfaces can change from one region to another or even from one road to another. Buildings and other structures are constantly in motion, yet the majority of these movements are imperceptible. Defects, ground movement, failed foundations, and deterioration of the building’s structure are all possible causes of movement. A structure is liable to crack if it cannot handle this movement. Landslides, vibrations, earthquakes, the deterioration of soft clay brick, chemical contaminant-induced concrete erosion, and other factors can all result in cracks. Buildings, bridges, roads, pavements, railroad tracks, cars, tunnels, aircraft, etc. can all fracture or split into two or more pieces, completely or partially. If left untreated, distortions and cracks can compromise the structure’s integrity, safety, and stability in addition to being unsightly and unsettling to the occupants.

Understanding the causes of cracking is necessary before any effective remedy can be applied. Then a plan for repair can be put into action. Currently, no automated system meets Tunisian requirements and conditions. That is why manual inspection in the laboratory remains the only solution. Often around the world, it remains the most widely used method as heavily textured pavements are increasingly common on global road networks. So, a reliable and automatic method of detecting these defects is the object of research of many teams around the world and more particularly in Tunisia. The maintenance of the road network requires knowing these degradations and their evolutions as soon as possible in order to repair them at a lower cost. Currently, in Tunisia, several thousand kilometers of national roads are examined visually each year. The idea is then to “bring the road to the office”, i.e., to eliminate visual input directly on the road at low speed and to gradually automate the work of road network agents, which consists of detecting and then classifying pavement surface damage. The pavements effectively crumble under the impact of excessive traffic and environmental variables. The various surface faults that appear are one of the state indicators of the evolution of the structure of these pavements. Road network maintenance demands early detection of these degradations and their development in order to lower the cost of repairs. Many countries currently visually examine thousands of kilometers of national roadways each year, which is both costly and time-consuming. Potholes may now be identified and categorized automatically thanks to machine learning algorithms and IoT technologies.

**Figure 1:**
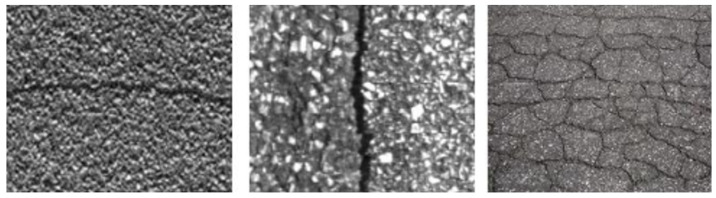
Some examples of real road cracks.

There are three basic reasons why automatic detection in pavement cracks is challenging: The size of the flaws to be recognized is rather small, the pavement texture varies significantly, and outside acquisition settings frequently include uncontrollable elements. It is important to remember that “crack” type faults are the most carefully scrutinized worldwide. These pavements do in fact have a large amount of background noise. The issue with this kind of pavement is that the cracks’ properties are, locally, quite similar to those of the texture, particularly of the intergranulate region. Many challenges arise while analyzing photos to find fractures in the pavement’s surface. In fact, uncontrollable elements such as humidity, fluctuation in the reflection coefficient, and even coating texture affect how the pavement appears in photographs. When creating such an analytical system, several elements should be considered. The issue of crack visibility, which typically presents a weak contrast with the texture of the pavement in which they are implanted, is added to this context. Hence, it is challenging to discern pavement fractures. In Tunisia, this type is the predominant defect. Images of Tunisian pavements are heavily textured as it is outlined in Figure 1. Indeed, these pavements contain a significant noise brought by the background texture.

In this work, we are particularly interested in road cracks. We treat cracks of different sizes, on different types of pavements. The focus of this work is on heavily textured pavements. To demonstrate the methods’ effectiveness, a sizable dataset needs to be used for training and testing machine learning algorithms. Following a survey of the literature, we attempt to find a technique that enables the preprocessing step to account for the crack’s shape. However, in order to distinguish the crack from noise, particularly that produced by the inter-aggregate space, we search for a more accurate model of the crack. This allows us to identify the class to which a given pavement image may belong. The extraction of their distinguishing features is made possible by the 3D reconstruction of the detected crack. The images were manually taken using a digital camera while we built our own database. Thus, the linear camera is thought to be the best option for acquiring dynamic images at high resolution.

The thresholding strategy provides the foundation for the approaches suggested for the binarization phase, which delimits the two classes present in the images (crack and background noise). Given the nature of the photographs, this procedure appears to be the best course of action. In addition, it is quick and cheap to implement in terms of calculation time. As the local thresholding findings nearly gave us the same threshold for various regions of the grayscale image, we chose the global strategy. The OTSU thresholding method produced good processing outcomes when applied to photos of low-noise pavements. The goal is then accomplished by separating the pixels that might be a part of a fracture from the background. The photographs we have been working on, however, are of severely textured pavement. We had no choice but to discover another thresholding method that was less noise-sensitive. The fuzzy C-threshold algorithm was employed by us (FCM).

The contributions of the work are enumerated in the following bullet points.

- Creating an actual dataset of heavily textural road cracks that may be used to train and test machine learning algorithms.
- Developing a technique for the automatic classification of cracks.
- Devising a method for 3D reconstruction of road cracks.

The manuscript is organized as follows. Section 2 reviews existing works of road crack detection. Section 3 details the proposed technique for identification, reconstruction, and classification. Section 4 describes the dataset and reports the efficiency of the technique. Finally, Section 5 concludes this work and provides directions for future work.

## 2. Related Works

Image processing-based techniques for crack detection have gained wide acceptance. This is due to the widespread accessibility of high-resolution cameras, including those found on smartphones [8] and drones [9]. Two popular methods are used: operation-based techniques and machine learning methods [10]. Earlier work developed techniques for crack segmentation using conventional techniques such as recursive tree-edge pruning with shadow removal [11], Gabor filter local binary pattern [12], morphological filters [13], and edge detection algorithms [14].

Machine learning algorithms have reemerged in the last decade as an effective method for handling computational science and data mining problems. This is primarily attributable to advancements in silicon technology, the availability of billions of data-gathering sensors, and the development of deep-learning methodologies as pointed out by Cubero-Fernandez, A., et al. in [15]. A solution to the traditional method’s drawback is crack detection using machine learning techniques. The method consists of four tiers: pre-processing, noise removal, and a collection of representative datasets. The labeling of datasets makes up the third tier. The machine learning model is trained in the fourth tier. Model testing is addressed in the final stage. Deep neural network (DNN)-based real-time crack detection has been proposed in Mandal, V., et al. [16]. The DNN’s accuracy was increased by the authors Chun, P.J., et al. [17] by retraining the network using incorrectly classified images.

In some circumstances, road images are corrupted by noise or can be of a low-quality which can lead to either false positives or false negatives (incorrect classification). To counter this issue, a two-stage CNN (convolution neural network) has been devised by Nguyen, N.H.T., et al. [18]. The first stage performs image denoising and incorporates any possible cracks in a narrow area. The second stage identifies the cracks. An automated system using drones and an optimized pre-trained CNN model (VGG-19) and linear SVM (support vector machine ) classified has been reported by Samma, H., et al. in [19] to detect road conditions. It has been suggested Hammouch, W., et al. in [20] to use GPS/DGPS (global positioning system/differential global positioning system) and three cameras mounted on a vehicle to detect longitudinal cracking and crocodile activity. With the aid of transfer learning, VGG19 detects, and categorizes cracks.

## 3. Proposed Approach

**Figure 2:**
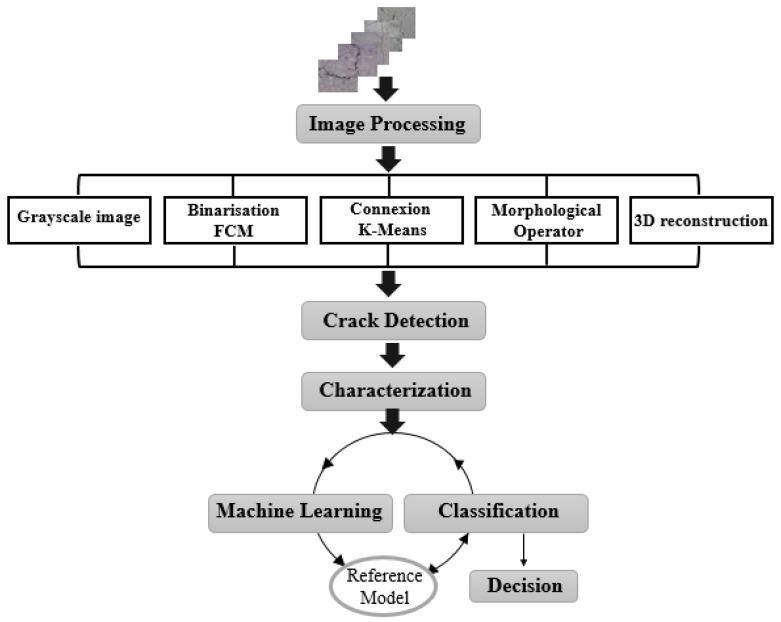
Block diagram of the crack detection and classification system.

In this section, we explain the different steps of our system of detecting and classifying cracks in pavements which are presented in the block diagram of Figure 2.

### 3.1. Image Processing

There are various challenges involved in image processing for the detection of pavement fractures. The difference in the reflection coefficient, the humidity, or even the roughness of the pavement all have an impact on how the pavement appears in the photographs. When creating such an analytical system, several elements should be considered. The issue of crack visibility, which typically presents a weak contrast with the texture of the pavement in which they are implanted, is added to this context. To solve these issues, we first suggest that our method begin with a pre-processing stage that includes grayscale conversion and image segmentation.

#### 3.1.1. Grayscale Conversion

The pixel at the (x,y) location is converted to the gray level using (1).

(1) Gray=0.2989×R+0.5870×G+0.1140×B

After the conversion stage, an image segmentation algorithm is used to separate the crack pixel from the background pixel.

#### 3.1.2. Images Segmentation

The segmentation process involves dividing the crack regions from the background noise regions. The challenge with this step is that the pavement images we will use have a lot of texture. The background texture in these images contributes a significant amount of noise. As a result, separation techniques must be used to enable the isolation of the two objects, crack and noise. Binarization and connection are the two components that make up the segmentation phase. The connection is based on the presumption that the crack is round, while binarization is based on the characteristic of the crack’s grayscale intensity.

**Figure 3:**
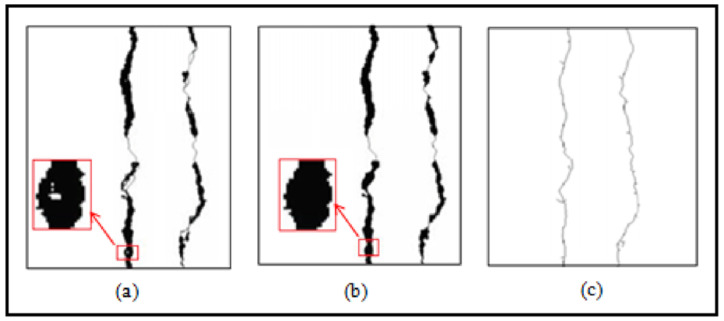
Principle of morphological filtering. (**a**) Binarized Image, (**b**) Small hole filling results, (**c**) Skeletonization result.

- Step 1—Binarization: The image from a grayscale scan has two types of information, the inhomogeneous texture of the backgrounds and the cracks which is weakly contrasted with respect to the texture. To delimit these two classes, we adopted the thresholding technique. The latter is used to reduce the information contained in the image to keep only the useful pixels that represent the objects of interest which are the cracks in our case. We propose the Fuzzy C-Means (FCM) thresholding algorithm [21]. It is an iterative classification method that classifies pixels according to C classes. It calculates each time the centers of the classes and generates the membership matrix *U* of the pixels in these classes.
- Step 2—Labeling of connected components: The second sub-step consists of grouping the pixels obtained at the binarization step in order to remove the noise and find the entire shape of the crack. This phase consists in extracting regions of connected pixels having common properties using the value of each pixel and the interactions with their neighboring.
- Step 3—Filtering by morphological operators: When there are no imperfections in the photos, the black areas are scattered throughout the picture at random. In the image, these areas appear as noise. We apply a morphological closure using the 8 × 8 disk structuring element in order to connect neighboring pieces of cracks. It is noted that the closure operation is expansion followed by erosion. Similarly, we reduced the thickness of the defect by a skeleton search using the “Thin” filter of size 3 × 3 to facilitate the extraction of parameters such as orientation for the characterization phase. Indeed, the skeleton reduces dimensions. Before that, it is essential to fill the small holes in the components in order to avoid noises on the skeleton. An illustration is given in Figure 3.

After completing this stage, we were able to join discrete crack segments and eliminate background noise in order to identify the complete crack shape. As a result, we obtain a binary image containing groups of black pixels on a white background.

**Figure 4:**
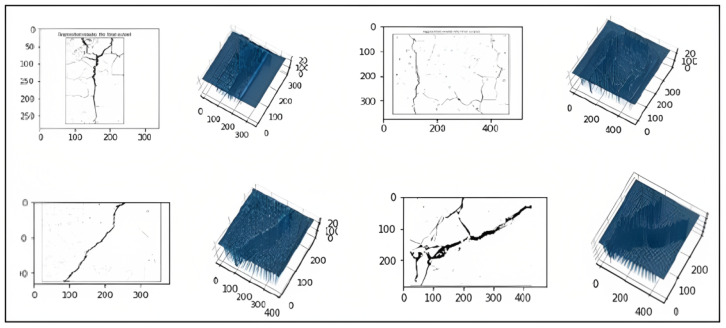
3D reconstruction of diverse types of cracks.

- Step 4—3D reconstruction: Image processing is an essential step to detect and extract the region of interest. Figure 4 presents an example of 3D crack reconstruction from the 2D processed image. The 3D representation of the image helps us to calculate the depth of the crack which is considered an important primitive. This characteristic is used to determine the type of crack if it is minor, moderate, or severe.

### 3.2. Region of Interest Detection

**Figure 5:**
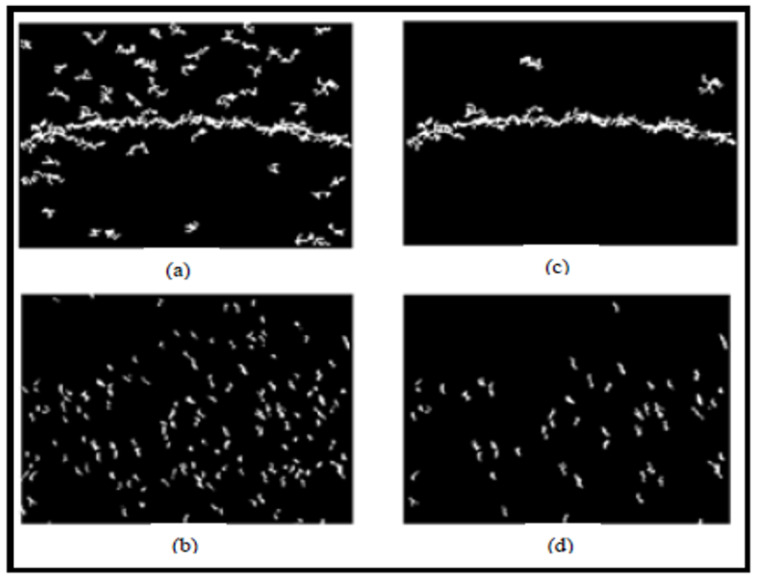
Region of Interest Extraction (**a**) Binarized transverse crack (**b**) Image without crack, (**c**,**d**) Noise suppression.

We started by deleting any locations less than 32 pixels from which a component will be regarded as a noise region since cracks can be fragmented. This is the ideal threshold that produced satisfactory results for most of the database’s photos, including those with and those without fractures. In order to detect the region of interest, which is the pavement crack, we were able to reduce the number of inconsequential size regions for both types of images with and without cracks, as shown in Figure 5b,d.

### 3.3. Characterization

Five attributes are extracted, four local (length, width, surface of a component, and attributes of projections) and two global (Hough attributes). These two global attributes allow us to determine if a related component is large enough to be directly considered as a crack.

#### 3.3.1. Length and Width

**Figure 6:**
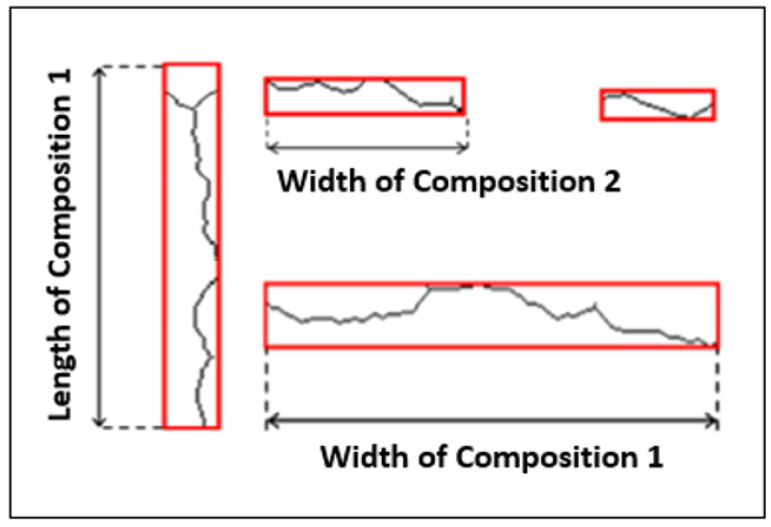
Length and width of the crack. The approximate size of the crack is depicted in the red box using a rectangle.

We have chosen the length and width of the bounding box of a component as the length and width of the region of interest as mentioned in Figure 6.

#### 3.3.2. Crack Severity

**Figure 7:**
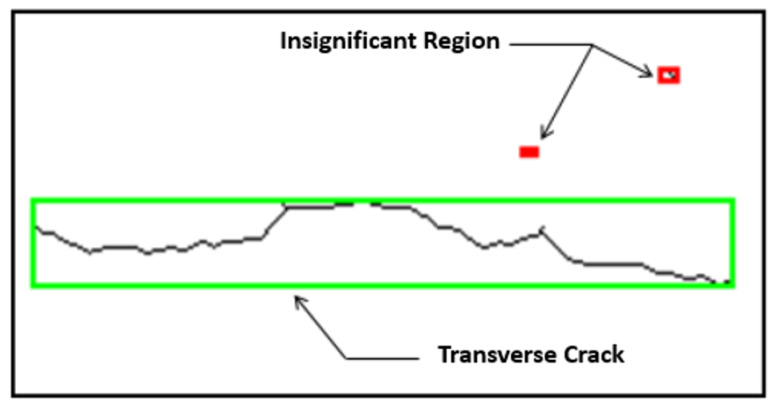
Crack severity.

This attribute is defined by calculating the ratio between the number of black pixels present in the region and the total number of black pixels describing the crack in the image (Figure 7). A ratio is used here for the purpose of normalizing the surface.

#### 3.3.3. Projection Attributes

**Figure 8:**
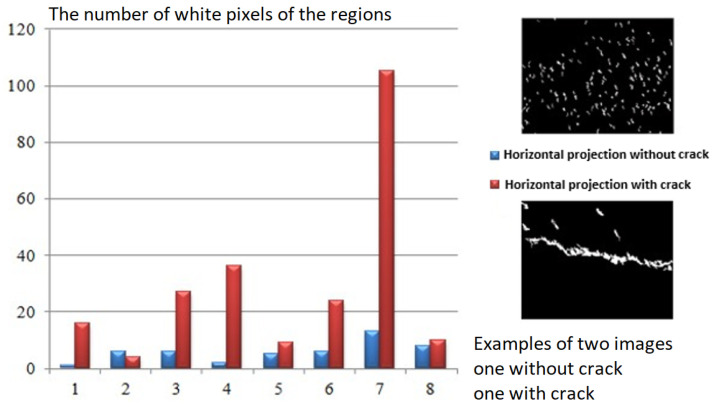
Determination of horizontal projection peaks.

**Figure 9:**
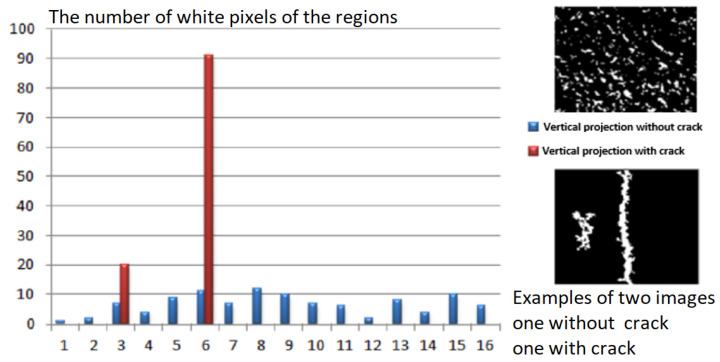
Determination of vertical projection peaks.

According to our observations, the cracks first emerge as discontinuities and subsequently as a series of minor “portions” of the crack. We also employed horizontal and vertical projections as features to identify the presence of cracks. Indeed, the distribution of the regions is random if the image does not contain a fracture. According to the various orientations, the resulting profiles do not exhibit particularly high peaks; however, if there is a fracture in the image, at least one of the profiles will appear to have a high peak. Hence, we identified the peak of the horizontal projection which is defined as the largest number of white pixels to measure the thickness of the component. While the height of the vertical projection determines the length. Figure 8 and Figure 9 show the number of white pixels of the regions obtained for two images with and without crack chosen from our database. The defect is clearly visible on the profile.

#### 3.3.4. Global Attributes in Hough Space

**Figure 10:**
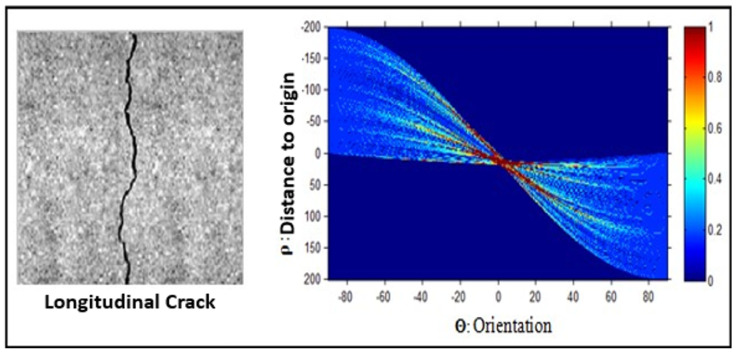
Hough Transformation for an example of crack.

The Hough transform [22] makes it possible to extract the global orientation attributes of the binary image as well as the entire length of the crack. The binary image obtained after the segmentation step is projected in several directions (in our case in the following 8 directions 0${}^{\circ }$, 22.5${}^{\circ }$, 45${}^{\circ }$, 67.5${}^{\circ }$, 90${}^{\circ }$, 112.5${}^{\circ }$, 135${}^{\circ }$, and 157.5${}^{\circ }$). We, therefore, apply a TSH to the binary image with an angular resolution θ of 22.5 degrees, and a distance resolution ρ=1. Figure 10 presents an example of the result of the Hough transforms applied to a longitudinal crack image.

**Figure 11:**
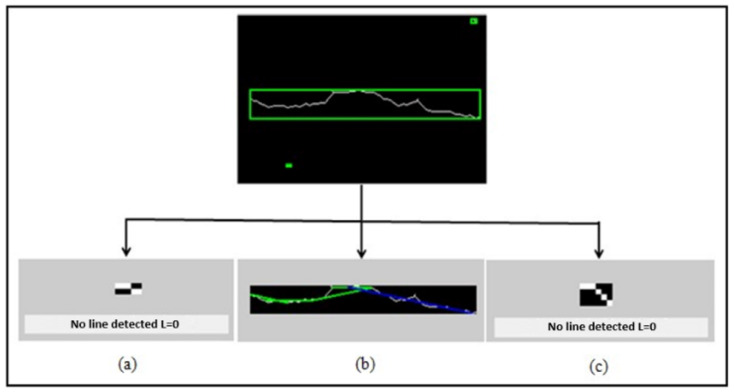
Choice of the best Hough line (**a**,**c**) No line detected, (**b**) Hough line. The green box illustrates the shape of the crack approximated using a rectangle.

The Hough transform result shown in Figure 10 reveals that the image contains a single crack due to a single large cluster in the image. It is also closer to 0${}^{\circ }$, which indicates the presence of a vertical (longitudinal) crack. The use of this type of descriptor allowed us to detect the presence of fine cracks in our pavement images; however, in the case of a very thick crack, its Hough transformation will propose several one-pixel-wide lines next to each other to actually represent the same line, but only one line is representative of this crack. This problem comes from the fact that the pixels representing a crack are rarely aligned and that if the straight line in question is several pixels wide, it actually corresponds to several straight lines. A search for the maxima of the lines of the Hough transform is used to overcome this problem and obtain signatures to determine the characteristics of the crack (line colored in blue in the Figure 11b.

The Hough attributes allow us to analyze the alignment at the global level since its representation completely preserves the original data of the image and also eliminates the noise; however, they remain difficult to implement for the analysis of attributes at the local level (on an area or on each related component), especially when the cracks are not very straight. Since the θ and ρ coordinates are represented in the *x* and *y* coordinates of the Hough transform, respectively, the position and orientation can be identified easily. Therefore, we determined the parametric values of the straight lines of each component based on the parameters of the Hough MA Accumulation Matrix (ρ,θ).

**Figure 12:**
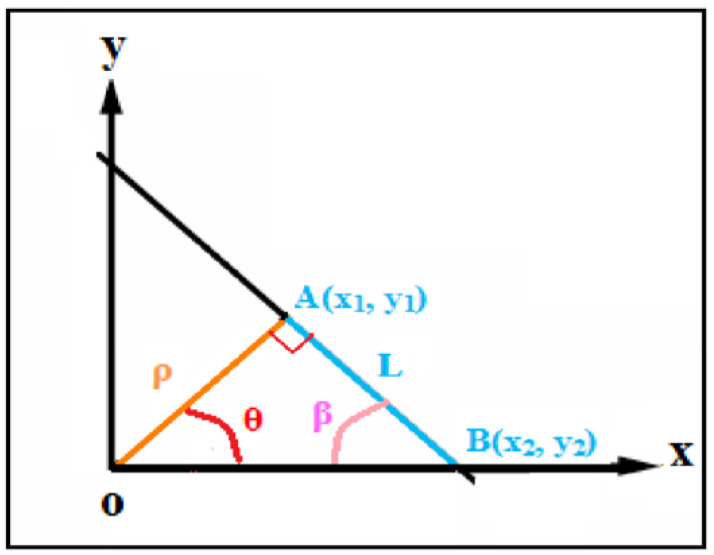
Determination of attributes by the Hough transform.

The length of the crack (L: length of the maximum Hough line) is defined as the length of the adjacent side of a rectangular triangle (OÂB) (Figure 12). The length is therefore determined by Equation (2)

(2) $$L=\sqrt{{\left(x_{1}-x_{2}\right)}^{2}+{\left(y_{1}-y_{2}\right)}^{2}}$$

To determine the value of the orientation of the crack (β), we started with the fact that the sum of the angles of the rectangle is equal to 180${}^{\circ }$. Then, we applied (3) with the angle of the maximum Hough line.

(3) $$\beta =\frac{\pi }{2}-\theta .$$

The determination of these two characteristics such as length and orientation by the Hough method allowed us to identify the crack and locate its location in the image.

#### 3.3.5. Parameter Normalization

To facilitate the recognition step, we first started by normalizing the parameters extracted during the characterization phase. As these primitives are initially expressed in pixels, it is, therefore, necessary to obtain standardized attributes in order to make them comparable to other values of the same domain to make them more significant and useful for the classification phase. To achieve this, we performed the normalization of the parameters of the characteristic vectors in the interval [0, 1] according to Equations (4)–(10) with *a* and *b* being, respectively, the height and the width of the binarized image, and *D* is the length of the diagonal of the image.

(4) $$\begin{array}{ccc} D & = & \sqrt{a^{2}+b^{2}} \end{array}$$

(5) $$\begin{array}{ccc} \mathrm{Height} & = & \frac{\mathrm{Height}\;\mathrm{bonding}\;\mathrm{box}}{a} \end{array}$$

(6) $$\begin{array}{ccc} \mathrm{Width} & = & \frac{\mathrm{Width}\;\mathrm{bonding}\;\mathrm{box}}{b} \end{array}$$

(7) $$\begin{array}{ccc} \mathrm{Surface}\;\mathrm{Region} & = & \frac{\mathrm{Number}\;\mathrm{of}\;\mathrm{white}\;\mathrm{pixel}\;\mathrm{in}\;\mathrm{the}\;\mathrm{region}}{\mathrm{Number}\;\mathrm{of}\;\mathrm{white}\;\mathrm{pixel}\;\mathrm{in}\;\mathrm{the}\;\mathrm{image}} \end{array}$$

(8) $$\begin{array}{ccc} \mathrm{Segment}\;\mathrm{length} & = & \frac{\mathrm{Maximum}\;\mathrm{length}\;\mathrm{projection}}{a} \end{array}$$

(9) $$\begin{array}{ccc} \mathrm{Segment}\;\mathrm{depth} & = & \frac{\mathrm{Maximum}\;\mathrm{horizontal}\;\mathrm{projection}}{b} \end{array}$$

(10) $$\begin{array}{ccc} \mathrm{Crack}\;\mathrm{length} & = & \frac{\mathrm{Length}\;\mathrm{of}\;\mathrm{the}\;\mathrm{maximum}\;\mathrm{Hough}\;\mathrm{line}}{D} \end{array}$$

**Figure 13:**
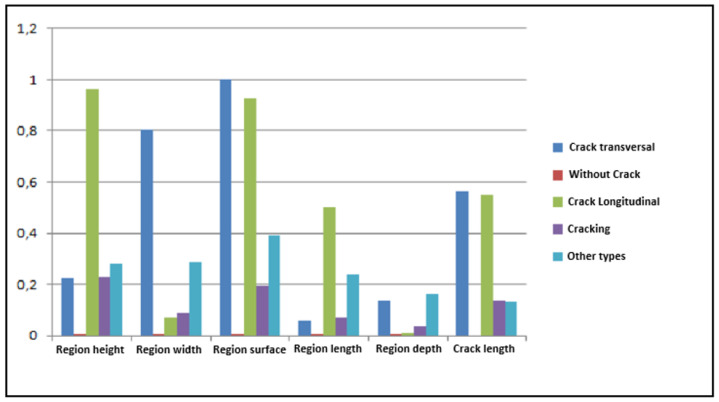
Variation of interclass primitives (X-axis: Primitives associated with each class of crack; Y-axis: Percentage).

**Figure 14:**
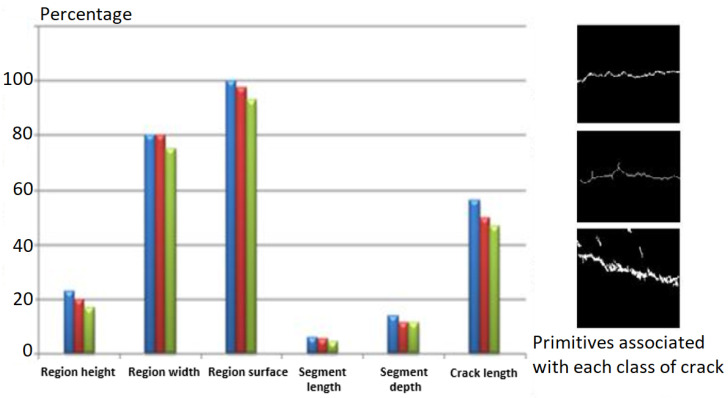
Variation of primitives for a transversal crack. The red, blue, and green colours represent three samples from the same transversal class to prove the intraclass variations of the primitives.

A morphological study of the different images from our database mentioned above shows that the primitives linked to each of the classes of cracks present a significant interclass variation (Figure 13) and a weak intraclass variation (Figure 14).

As shown in Figure 13, the attributes are characterized by very high normalized attribute values close to 1 that represents the crack regions while the other regions, i.e., those characterized by low relative parameters (the closest to 0) are considered to be a background texture (noise).

### 3.4. Machine Learning and Crack Classification

**Figure 15:**
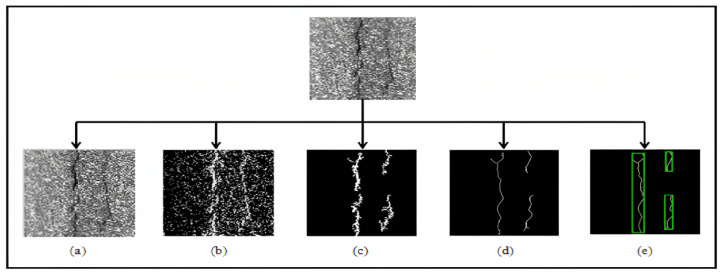
Location of the crack: (**a**): Original image; (**b**): Binarized image; (**c**): Denoising of the binarized image; (**d**): Skeletonization; (**e**): Crack detection.

The classification consists of assigning a given crack shape to one of the predefined classes. In our case, the cracks are classified as longitudinal, transverse, cracking type, or other shapes, depending on the primitives previously extracted. This recognition phase combines the two tasks of learning and decision. Learning involves automatically the decision rules based on a set of already classified examples. The outcome of the decision is an “opinion” on whether the form belongs to one of the classes based on the learning models. The classification phase is highly dependent on the result of the segmentation, especially on the importance of the noise at this stage and the degree of discontinuity of the segmented cracks. In reality, these crack portions are considered to be continuous cracks (real image). Whereas in practice, the cracks present discontinuities which translate that the crack is a set of portions of cracks (Figure 15e).

Considering the fact that the segmented components can be continuous or not in the resulting image of the segmentation, we have divided the classification operation into two levels. A step concerns the characterization of the defect, that is to say, to specify the type of the connected component and a step specifies the type of the crack. To achieve this, we used the SVM approach for the two classification sub-steps [23]. The benefit of using the SVM algorithm to select such a decision function is that the resulting solution corresponds to the convex function’s optimum. With a wide choice of kernels, SVMs allow great freedom in the form of classes (with control by regularization). It, therefore, does not have several local optima as for neural networks (in their classical formulation), but a global optimum. This optimum corresponds to the minimization of the structural risk and therefore to the search for a hypothesis with good generalization capacities from a given space of hypotheses. In addition, the space assumptions depend on the choice of the kernel function. This method is also characterized by a very fast learning method with a relatively limited number of examples provided by the relevance check. In addition, it is also less sensitive to the imbalance between positive and negative examples. For each image of cracks in the database, we define an SVM architecture to which we teach both the good and the bad answers (among the whole of the supervised learning database). The system learns all the vectors of primitives of the images chosen for the learning phase. Once trained, the model thus constructed makes it possible to decide whether to belong to one class rather than another for any new pavement image submitted to the system. Once our training and validation files have been built, the only parameters to set remain those of the SVMs. Thus, we applied our algorithm following a preliminary study and practical considerations, such as:

- Due to the number of support vector machines to manage and the number of classifiers to estimate, we opted for the “one against all” classification strategy which allows us to manage a minimal number of classifiers.
- We opted for the Gaussian kernel RBF (radial basis function) as it is the kernel frequently used in the literature and which has demonstrated the best performance in terms of pavement image classification. The kernel parameter σ was set to 6 (this value was selected experimentally to provide the optimum accuracy and performance).
- The trade-off C is used to fix the trade-off between minimizing the learning error and maximizing the margin. The higher the value of C, the more the capacity of the classifier is optimal. In our case, the value of C has been fixed in a heuristic way. We opted for a value of C = 1000. To accelerate the learning of SVMs and improve their performance, we used the SMO (sequential minimal optimization) method. Indeed, the SMO algorithm segments the initial optimization problem into sub-problems for which we have an analytical solution. Its implementation is easy to implement since it does not require the use of a particular optimization library.

The system learns all the vectors of primitives defined for each component then it will predict the classification results of the regions according to the learning base created. The next step is used to specify the nature of the crack (transverse, longitudinal, cracking, etc.) in the overall image. To achieve this, we determined new characteristics based on the results obtained at the end of the first classification step. Indeed, for each image of our database, we determined the proportions of the appearance of the classes of the regions in the totality of the image. We then defined four parameters $P_{1}$, $P_{2}$, $P_{3}$, and $P_{4}$ determined using Equations (11)–(14). $P_{1}$, $P_{2}$, $P_{3}$, and $P_{4}$ represent, respectively, the proportions of regions without cracks, horizontal, vertical, and other cracks in the input image and *N* defines the total number of regions in the image.

(11) $$\begin{array}{ccc} P_{1} & = & \frac{\mathrm{Number}\;\mathrm{of}\;\mathrm{regions}\;\mathrm{classified}\;\mathrm{free}\;\mathrm{of}\;\mathrm{cracks}}{N} \end{array}$$

(12) $$\begin{array}{ccc} P_{2} & = & \frac{\mathrm{Number}\;\mathrm{of}\;\mathrm{regions}\;\mathrm{with}\;\mathrm{horizontal}\;\mathrm{cracks}}{N} \end{array}$$

(13) $$\begin{array}{ccc} P_{3} & = & \frac{\mathrm{Number}\;\mathrm{of}\;\mathrm{regions}\;\mathrm{with}\;\mathrm{vertical}\;\mathrm{cracks}}{N} \end{array}$$

(14) $$\begin{array}{ccc} P_{4} & = & \frac{\mathrm{Number}\;\mathrm{of}\;\mathrm{regions}\;\mathrm{with}\;\mathrm{cracks}\;\mathrm{with}\;\mathrm{other}\;\mathrm{orientation}\;}{N} \end{array}$$

**Figure 16:**
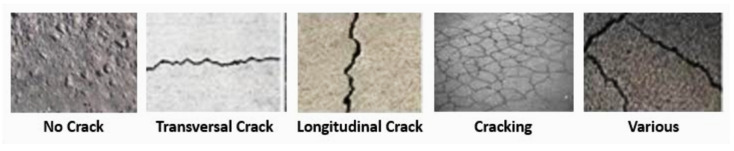
An example of a crack for each class.

At this stage, we also adopted the SVM technique to classify the crack images into the five classes described in Table 1. An example of a crack for each class is presented in Figure 16.

Our system will predict the classes to which an image belongs based on the learning models. At the end of this classification phase, we succeeded in arranging the test images by assigning each of them to the most appropriate class. We applied our two-level classification approach on the different test images.

## 4. Experimental Results

### 4.1. Dataset

**Figure 17:**
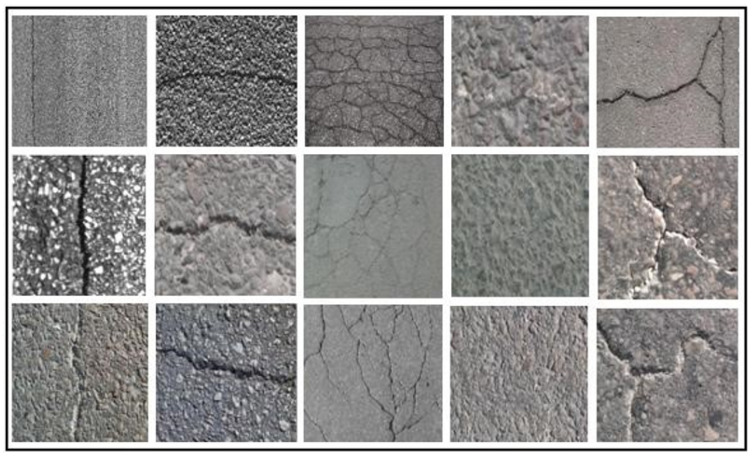
Examples of images from our dataset obtained in static mode.

The proposed system was tested by our own dataset. It contains 330 real pavement images divided into five classes as shown in Table 2. Some examples of images are shown in Figure 17.

### 4.2. Execution and Learning Times

The average execution time is as follows (for a real image of size 640 × 480 pixels):

- Crack detection time: 998 ms,
- Crack characterization time: 331 ms, item Region classification time: 393 ms (depending on the number of regions),
- Crack classification time: 59 ms,
- Total: 1781 ms (18 s).

Learning times are:

- Region learning time: 421 ms (depending on the number of regions),
- Crack learning time: 171 ms.

### 4.3. Evaluation of the Crack Detection Phase

**Figure 18:**
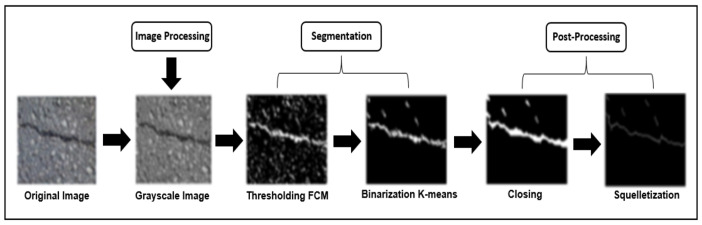
Crack detection system.

Figure 18 presents the different processing applied to the inspected image to detect the crack and remove the noise of the background texture. The full valuation, based on different images in the dataset, is shown in Table 3.

According to the results shown above, the method has been successful in localizing the fracture in the majority of static picture acquisitions. By localization, we mean that we are able to tell the difference between the crack and the bottom of the image and false alarms. Images having a texture that is roughly smooth are best for our purposes because they have the lowest false detection rates. However, for the hardest photos to analyze, the probability of false detection is larger. There is a significant texture in these pictures. The issue of items (aggregates) that do not match the fracture but appear with gray levels close to those of the cracks is another issue in these photographs, as well as in all images.

### 4.4. Performance of the Crack Classification Phase

A thorough understanding of the retrieved primitives is necessary for the decision. This phase is used to evaluate the performance of the system and to determine the overall recognition rate of our detection system. The first interesting criterion for road network managers is whether a portion of the road contains cracks or not, without necessarily knowing their type. The database contains 330 pavement images of which 165 training images are used to construct the classifier and the rest of the images in the database are used to test the classifier and estimate its actual error rate.

The following table shows the percentages of defects detected on both sets of images with and without cracks. The overall percentage of error gives the proportion of misclassified images, however, it is the proportion of undetected defects that must be minimized to ensure that no defect images are ruled out during the pavement repair phase by road managers. The results of classification by the SVM approach are presented in Table 4.

Table 5 shows the classification results of the overall images, we see that the cross-sectional cracks are very well classified. Longitudinal cracks are less well detected with 18.2% non-detection, possibly due to the common labeling with cracking. The results on flawless images give the highest recognition rate with an error rate equal to 7.3%.

Following the implementation of our approach, we have achieved our objective of detecting and classifying crack-type defects in pavement images. To measure the quality of our system and evaluate the robustness of the classifier, we determined the confusion matrix for the five crack classes. The results obtained by this matrix are reported in Table 6.

As we see in Table 6, images without cracks are very well ranked with a good recognition rate (TBR) equal to 92.68%. Whereas, images of transverse and longitudinal cracks are less well detected with lower good detection rates, respectively (90.48% and 81.82%). For the “earthing” and “other” classes, it is clear from the confusion matrix that these images are classified as “longitudinal” or “transverse” because of the existence of the majority of longitudinal or transverse cracks in these images.

**Figure 19:**
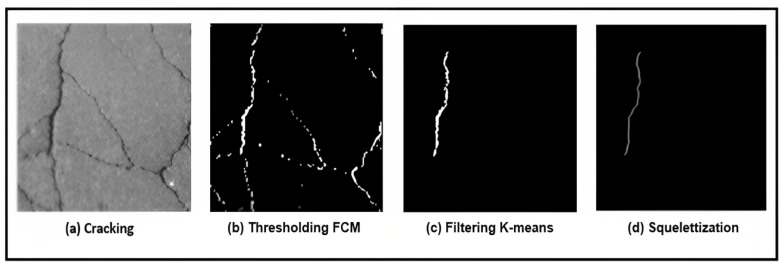
Example of false classification of cracking defect.

In addition, our system makes strong confusion with a rate of 50% between the images of cracking type and the images of longitudinal cracks. Figure 19 shows an example of the processing results for a cracking image. Figure 19c clearly demonstrates the strong confusion between the cracking class and the longitudinal crack class. This confusion may be due to the common labeling between these two types of classes.

Indeed, the result of extraction of relevant primitives of these two classes gives a strong resemblance to the level of length and width of the segments constituting the crack as well as the orientation. The results on these types of defects show the limits of our method because the cracking types are not well localized. This type of problem can be solved by eliminating the K-means filtering phase for only this class.

**Figure 20:**
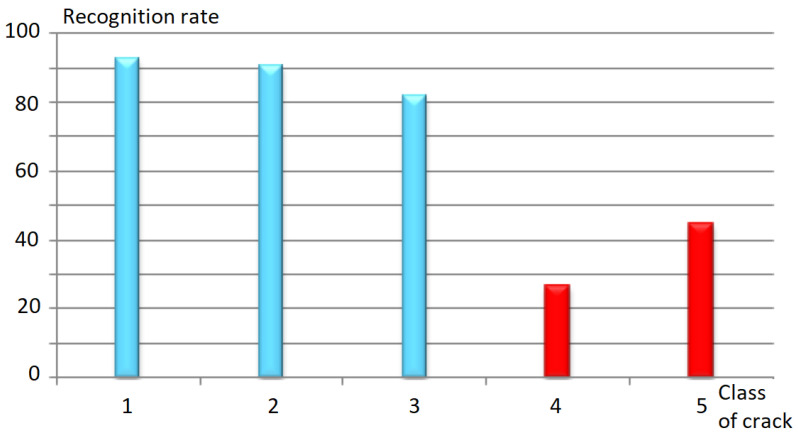
Recognition rate for each class of crack.

The recognition rate for each class is shown in Figure 20. Following the implementation of our proposed approach, we recorded an honorable recognition rate. We obtained promising results compared with the results found in the literature. Table 7 compares the performance of the proposed method to recent ones, in which NA means not available.

## 5. Conclusions

We succeeded in developing a new method for the automatic detection of cracks on different types of pavement, especially on heavily textured pavements. This is a big challenge because it is a problem detecting a very thin object on a noisy background. We started our approach by converting the original image into a grayscale image. Then, the binarization of the grayscale image is performed using the FCM (fuzzy C-means) thresholding method. After that, we extracted the regions belonging to a crack from the classification of the latter using the K-means method. Then we extracted the relevant characteristics which make it possible to describe the whole shape of the crack in order to classify the cracks according to their types. We used the shape feature to describe the crack. We have chosen the length and width of the bounding rectangle of a component as the length and width of the crack. We also used horizontal and vertical projections to determine the severity of the defect. A crack is characterized by its orientation. The extraction of rectilinear structures and therefore of cracks is then obtained by applying the Hough transform. At the recognition level, an approach based on SVMs was adopted and an RBF-type kernel was retained. Our approach was validated on a database containing 330 pavement images showing the different types of cracks.

Improvements are possible for these methods, especially for the detection of cracks, by replacing the global thresholding method with a two-level binarization method. Other possible improvements to the presented work include:

- Increasing the size of our database to improve processing results.
- Taking into account a local threshold determined at the level of each region of the image instead of a global threshold applied to the image. This type of thresholding can solve the problem of false detection and thus allows the detection of other types of degradations in addition to cracks.

## Figures and Tables

**Table 1:** Crack classes.

Crack Class	Label
No Crack	1
Transversal Crack	2
Longitudinal Crack	3
Cracking	4
Various	5

**Table 2:** Dataset Composition.

Images without cracks	82
Images with transversal cracks	86
Images with longitudinal cracks	85
Images with cracking	60
Images with diverse cracks	17
Total	330

**Table 3:** Results obtained on sets of images with or without cracks.

Dataset	Rate of Crack Detection	Rate of False Detection
Images with crack	79%	21%
Image without crack	11%	89%
Detection percentage	84%	
False Detection percentage	16%	

**Table 4:** Results of classification.

Dataset	Percentages of Images Classified with Cracks	Percentages of Images Classified without Cracks
Images with crack	98.4%	1.6%
Images without crack	7.32%	92.68%
Error percentage	4.46%	
Recognition percentage	95.54%	

**Table 5:** Results obtained for different classes of the images.

Class of the Image	Number of Images	Error	Percentage of Images Classified Correctly	Percentage of Images Classified Correctly
1: Without crack	41	3	92.7%	7.3%
2: Transversal crack	42	4	90.5%	9.5%
3: Longitudinal crack	44	8	81.8%	18.2%
4: cracking	30	22	26.7%	73.3%
5: Other types	9	4	55.6%	44.4%
Total	166	41	75.3%	24.7%

**Table 6:** Confusion matrix of the classification of crack images.

Class	1	2	3	4	5
1 (Without Crack)	92.68	2.44	2.44	2.44	0.00
2 (Transversal Crack)	0.00	90.48	2.38	7.14	0.00
3 (Longitudinal Crack)	2.27	6.82	81.82	9.09	0.00
4 (Cracking)	3.33	20.00	50.00	26.66	0.00
5 (Other Types)	0.00	22.22	22.22	11.11	44.44

**Table 7:** Comparison with recent works.

Reference	Dataset	Classification	3D Reconstruction	Run-Time	Accuracy
[17]	Private-3D mobile mapping system	CNN	No	NA	94%
[18]	Public and private	two-stage CNN	No	NA	91%
[19]	private and SDNET2018 (public)	pre-trained VGG-19	No	NA	96.4%
[20]	Private	CNN	No	NA	93.45%
This work	private	SVM	Yes	51 ms	95.54%

## Data Availability

The data presented in this study are openly available in https://figshare.com/s/43672f610611d2b269fa (accessed on 12 January 2023).

## Abbreviations

The following abbreviations are used in this manuscript:

DNN	Deep-Neural Network
CNN	Convolution Neural Network
SVM	Support Vector Machine
GPS	Global Positioning System
DGPS	Differential Global Positioning System
FCM	Fuzzy C-Means
SMO	Sequential Minimal Optimization
NA	Not Applicable
